# Random forest model analysis of influencing factors of chemotherapy-induced peripheral neuropathy in patients with nasopharyngeal carcinoma: a cross-sectional survey study

**DOI:** 10.3389/fonc.2025.1548742

**Published:** 2025-07-18

**Authors:** Yanxin Zhang, Xiaojun Huang, Guirong Yang, Wei Li, Limin Liang, Lina Wei, Jiamei Lu

**Affiliations:** Radiotherapy Department of the First Affiliated Hospital of Guangxi Medical University, Nanning, Guangxi, China

**Keywords:** random forest model, nasopharyngeal cancer, chemotherapy-induced peripheral neuropathy, cross-sectional study, symptom management

## Abstract

**Background:**

Chemotherapy-induced peripheral neuropathy (CIPN) is a progressive and persistent condition that can significantly affect the quality of life of patients. Even after the cessation of chemotherapy, symptoms of CIPN may endure for an extended period. Despite its considerable impact, there is a paucity of research examining the effects of CIPN on patients.

**Objective:**

To investigate the current status of CIPN in patients with nasopharyngeal carcinoma (NPC) and accurately identify factors influencing neurotoxicity based on the Random Forest algorithm.

**Method:**

A total of 289 patients with NPC were admitted to the Radiotherapy Department of the First Affiliated Hospital of Guangxi Medical University between August 2023 and April 2024 to investigate the current status of neurotoxicity, cognitive impairment, fatigue, and hand-foot syndrome. To rank the importance of the influencing factors of CIPN in NPC patients, a random forest model was constructed to analyze the influencing factors.

**Results:**

The incidence of CIPN in NPC patients was 81.7%. The results of the random forest algorithm showed that age, chemotherapy cycle, tumor stage, chemotherapy regimen, concurrent chemoradiotherapy, BMI, hand and foot syndrome fatigue, and cognitive dysfunction were the main factors affecting CIPN in NPC patients (P<0.05).

**Conclusion:**

There is an urgent need to accurately identify the risk factors for CIPN in patients with NPC and develop multidisciplinary collaborative prevention and intervention measures to achieve effective prevention and comprehensive management.

## Introduction

The incidence of nasopharyngeal carcinoma (NPC) in China ranks first in the world, and radiotherapy-based comprehensive chemoradiotherapy is the primary treatment (1). With the wide application of traditional chemotherapy, With the wide application of traditional chemotherapy, targeted and immunosuppressive drugs, adverse reactions such as heart, liver, and kidney function damage, bone marrow suppression, and gastrointestinal reactions are more common. Chemotherapy-induced peripheral neuropathy (CIPN) can lead to the change or destruction of patients’ movement, touch, pain, position sense, and vibration sense, as well as symptoms of nerve function disorders, such as numbness of the hands and feet, muscle weakness, and proprioception loss, which are progressive and lasting. Even after the cessation of chemotherapy, CIPN symptoms can persist for a long time, with an incidence of up to 60–80%, which has a serious impact on the social life and treatment rehabilitation of patients (2). In recent years, there have been few studies of CIPN symptoms. On one hand, patients lack relevant knowledge of CIPN symptoms; on the other hand, the impact of CIPN symptoms on patients’ daily functions can be easily ignored in the clinical work of medical staff (3). Therefore, this study was based on the random forest model to explore the current situation of CIPN in NPC patients, which is helpful to accurately identify the influencing factors of CIPN in NPC patients and provide a reference for clinical medical staff to formulate targeted interventions.

## Methods

### Study participants

A total of 289 NPC patients admitted to the Department of Radiotherapy, the First Affiliated Hospital of Guangxi Medical University, from August 2023 to April 2024, were selected as research subjects. Inclusion criteria were as follows: (1) confirmed by histopathology, received chemotherapy for the first time, and completed at least one chemotherapy cycle; (2) age > 18 years; (3) clear awareness, primary school education, or above, informed consent, and willingness to cooperate; exclusion criteria: (1) intellectual deficiency, mental disorders(examples include schizophrenia, delusional disorder, schizoaffective disorder, etc.), cognitive or consciousness disorders; (2) other malignant tumors or other serious systemic diseases; (3) peripheral neuropathy before chemotherapy; (4) Withdrawal and inability to cooperate with the researcher for various reasons. This study was approved by the Institutional Review Board of the First Affiliated Hospital of Guangxi Medical University (Approval No. 2023-S913-01).

### Sample size estimation

According to the sample size formula n=u_α_^2^P_0_(1-P_0_)/δ^2^, concerning the research of Liruolin et al. (4), the allowable error is 5%, α =0.05, bilateral u α =1.96, and the estimated sample size is at least 136 cases.

### Chemotherapy method

All patients received concurrent chemotherapy or induction chemotherapy. Details are presented as follows: (1) induction chemotherapy: GP (gemcitabine [1000 mg/m^2^ on days 1 and 8] and cisplatin [80 mg/m^2^ on day 2] every three weeks, for three cycles; or Taxel [260 mg/m2 on day 1] and cisplatin [75 mg/m^2^ on day 2] every three weeks, for three cycles; PF scheme: 5-FU 1000 mg/m^2^/24 hours, continuous pumping for 4 days; (2) concurrent chemotherapy: cisplatin-based concurrent chemotherapy, mainly with 80 mg/m^2^ on day 2 every three weeks, for two cycles.

### Instrument

#### General information questionnaire

Including patients’ general demographic data such as age and sex, and disease-related data such as treatment mode and disease stage.

#### Functional assessment of cancer therapy-cognitive function, FACT-Cog

Compiled by Lai et al. (5) and compiled by Xupeirong et al. (6) in 2023, it was used to evaluate the cognitive function of cancer patients, and the Cronbach’s α coefficient was 0.876. The scale includes four dimensions and 37 items: perceived cognitive impairment (20 items), perceived cognitive ability (9 items), cognitive evaluation (4 items), and impact on life (4 items). The Likert 5-level scoring method was adopted, and 0-4 points were assigned from “never” to “several times a day.” The total score is 0-148, and 75 was the critical value. The higher the score, the better the cognitive function of the patients.

#### Chinese version of the brief fatigue inventory, BFI-C

Compiled by Chang et al. (7) and Gaoliping et al. (8) in 2009, Cronbach’s α coefficient was 0.944. It is a one-dimensional scale with nine items in total. From “no fatigue to” severe fatigue, “it is assigned 0-10 points, with a total score of 0-90 points. The average score of the items is divided into: 0-3 points for mild fatigue, > 3-6 points for moderate fatigue, and > 6-10 points for severe fatigue.

#### Hand-foot syndrome, HFS

Hand-foot syndrome was graded according to adverse reaction evaluation criteria (9). Grade 0: No clinical symptoms or signs. Grade I: numbness or paresthesia of the hands and feet; erythema and vascular dilation of epidermal reticular tissue can be observed. Grade II: discomfort when holding or walking, painless swelling or erythema, and redness may also occur. Grade III: painful erythema and swelling of palms and soles, erythema and swelling around nails, chapped skin, and isolated necrotic keratinocytes on the epidermis. Grade IV: desquamation, ulcer, blisters, severe pain, and complete epidermal necrosis. HFS is diagnosed when the patient has grade I or higher hand and foot skin or sensory changes.

#### Functional assessment of cancer therapy/gynaecologic oncology group, neurotoxicity, FACT/GOG-Ntx

Compiled by Calhoun et al. (10) and Cheng et al. (11), it is widely used to assess the severity of CIPN symptoms in cancer patients, and the Cronbach’s α coefficient was 0.879. The scale consists of four dimensions and 11 items, including sensation (four items), hearing (two items), movement (three items), and dysfunction (two items). The Likert 5-level scoring method was used to assign 0-4 points to “never” and “very much.” The total score on the scale was 0-44 points. CIPN symptoms were present if the total score was > 0. The higher the score, the more serious the neuropathy.

### Research data collection methods

Conduct unified guidance and training for researchers, and inform the research object of the content and purpose of this study. After obtaining consent from the research subject, the researchers will uniformly issue a questionnaire in the ward, which will be filled in by the research subject, while avoiding the treatment and rest time of patients (survey time: 9:00-11:00 in the morning; 3:30-5:30 in the afternoon). A total of 300 questionnaires were sent out and 289 were recovered, with an effective recovery rate of 96.3%.

### Statistical processing

The data were checked and entered by two people in Excel, and the data were statistically analyzed using SPSS software (version 26.0). Quantitative data are expressed as mean ± standard deviation; qualitative data are expressed as the number of cases and constituent ratio (%). Pearson correlation analysis was used for correlation analysis, and the random forest classifier algorithm of R 4.2.2 software was used to construct a random forest map. With a test level of a=0.05.

## Results

### Univariate analysis of general information and CIPN in patients with NPC

Univariate analysis showed that the CIPN group and the normal group (taking the total score of neurotoxicity > 0 as the CIPN group) in patients with NPC had statistically significant differences in age, education level, payment method of medical expenses, chemotherapy cycle/stage, tumor stage, BMI/(kg/m ²), chemotherapy regimen, myelosuppression grade, and hand-foot syndrome (all p<0.05), which were the main influencing factors of CIPN. (See **Table 1**).

**Table 1:** Comparison of general data between the CIPN group and the normal group.

Project	Classification	Number of cases	CIPN group (236 cases)	Normal group (53 cases)	Statistical value	*P*
Age/year	18-	47	37 (19.9)	10 (18.9)	-2.505^a^	0.012
	31-	124	113 (47.9)	11 (20.8)		
	46-	84	64 (27.1)	20 (37.7)		
	56-	34	22 (9.3)	12 (22.6)		
gender	male	205	163 (69.1)	42 (79.2)	2.174^b^	0.140
	female	84	73 (30.9)	11 (20.8)		
marital status	married	199	168 (71.2)	31 (58.5)	4.071^b^	0.131
	unmarried	59	43 (18.2)	16 (30.2)		
	Divorced/widowed	31	25 (10.6)	6 (11.3)		
Educational level	Primary school and below	129	116 (49.1)	13 (24.5)	-2.666^a^	0.008
	middle school	58	43 (18.1)	15 (28.3)		
	High school/technical secondary school	56	40 (16.9)	16 (30.2)		
	College degree or above	46	37 (19.9)	9 (17.0)		
Place of residence	town	127	105 (44.5)	22 (41.5)	0.156^b^	0.693
	rural area	162	131 (55.5)	31 (58.5)		
medical insurance	Medical insurance for urban residents	178	139 (58.9)	39 (73.6)	9.549^b^	0.008
	Employee medical insurance	87	80 (33.9)	7 (13.2)		
	other	24	17 (7.2)	7 (13.2)		
Household per capita monthly income/yuan	<3000	134	111 (47.1)	23 (43.4)	-0.502^a^	0.615
	3000-6000	100	81 (34.3)	19 (35.8)		
	>6000	55	44 (18.6)	11 (20.8)		
Chemotherapy cycle/phase	1∼2	102	92 (38.9)	10 (18.9)	-3.75^a^	<0.001
	3∼5	114	95 (40.2)	19 (35.8)		
	>5	73	49 (30.9)	24 (45.3)		
Tumor stage	Phase II and below	51	46 (19.5)	5 (9.4)	-2.079^a^	0.038
	phase III	112	93 (39.4)	19 (35.8)		
	Phase IV and above	126	97 (41.1)	29 (54.7)		
BMI/ (kg/m²)	<18.5	126	91 (38.6)	35 (66.0)	-3.847 ^a^	<0.001
	18.5-24	99	85 (36.0)	14 (26.4)		
	>24	64	60 (25.4)	4 (7.5)		
Chemotherapy regimen	Pure Platinum	89	83 (35.2)	6 (11.3)	13.103^b^	0.004
	TP programme	81	65 (27.5)	16 (30.2)		
	DP programme	61	44 (18.6)	17 (32.1)		
	PF programme	58	44 (18.6)	14 (26.4)		
Concurrent chemoradiotherapy	Yes	47	31 (13.1)	16 (30.2)	9.242^b^	0.002
	No	242	205 (86.9)	37 (69.8)		
Myelosuppression grade	0	112	81 (34.3)	31 (58.5)	-3.030 ^a^	0.003
	I	80	68 (28.8)	12 (22.6)		
	II	56	52 (22.0)	4 (7.5)		
	III	28	25 (10.6)	3 (5.7)		
	IV	8	6 (2.5)	2 (3.8)		
	V	5	4 (1.8)	1 (1.9)		
HFS Grade	0	52	32 (13.6)	20 (37.7)	-2.674 ^a^	0.007
	I	88	75 (31.8)	13 (24.5)		
	II	91	81 (34.3)	10 (18.9)		
	III	44	37 (15.7)	7 (13.2)		
	IV	14	11 (4.6)	3 (5.7)		

TP scheme, Paclitaxel + platinum; DP regimen, Docetaxel + platinum; PF regimen, fluorouracil + platinum.

^a^= *Z*, ^b^=χ2.

HFS: 1)Grade 0: No clinical symptoms or signs. 2)Grade I: numbness or paresthesia of the hands and feet; erythema and vascular dilation of epidermal reticular tissue can be observed. 3)Grade II: discomfort when holding or walking, painless swelling or erythema, and redness may also occur.4) Grade III: painful erythema and swelling of palms and soles, erythema and swelling around nails, chapped skin, and isolated necrotic keratinocytes on the epidermis.5) Grade IV: desquamation, ulcer, blisters, severe pain, and complete epidermal necrosis.

### Current situation of fact/gog NTX, FACT-Cog, brief fatigue, and hand-foot syndrome in NPC patients

The total score of fact/Gog NTX in NPC patients was 22 (15, 28), with an incidence of 81.7%; the total score of cognitive impairment was (72.87 ± 18.48), and the overall incidence was 64.0%. The scores of each dimension from high to low were impacted on life, perceived cognitive impairment, perceived cognitive ability, and cognitive evaluation; The average score of BFI-C was (3.31 ± 0.86), and the overall BFI-C level was moderate; The incidence of HFS was 70.9%.

### FACT/GOG-Ntx and hand-foot syndrome、FACT-Cog、brief fatigue inventory relationship heatmap

**Figure 1:**
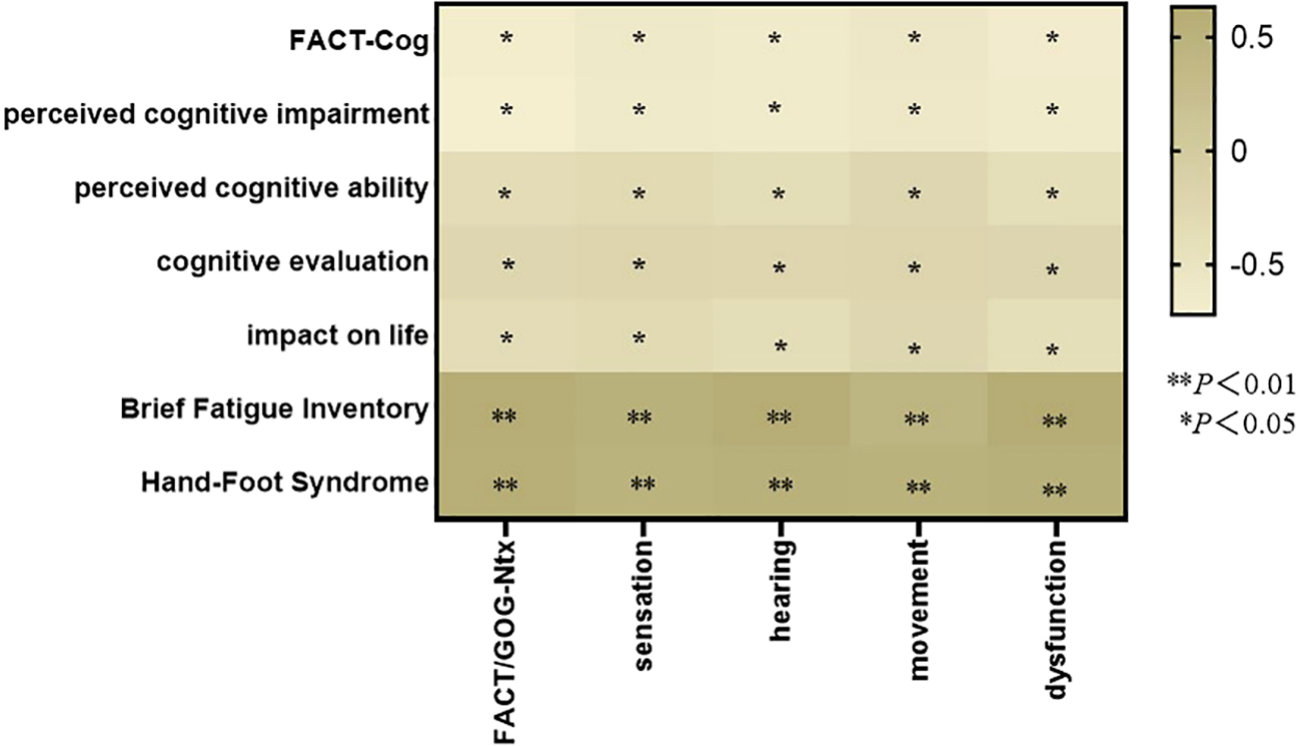
ACT/GOG-Ntx and Hand-Foot Syndrome、FACT-Cog、Brief Fatigue Inventory Relationship Heatmap.

Spearman correlation analysis showed that fact/Gog NTX was negatively correlated with fact-cog score (*P* < 0.05); Fact/Gog NTX was positively correlated with BFI-c score *(P* < 0.05). (See **Figure 1**).

### Random forest modeling and evaluation

**Figure 2:**
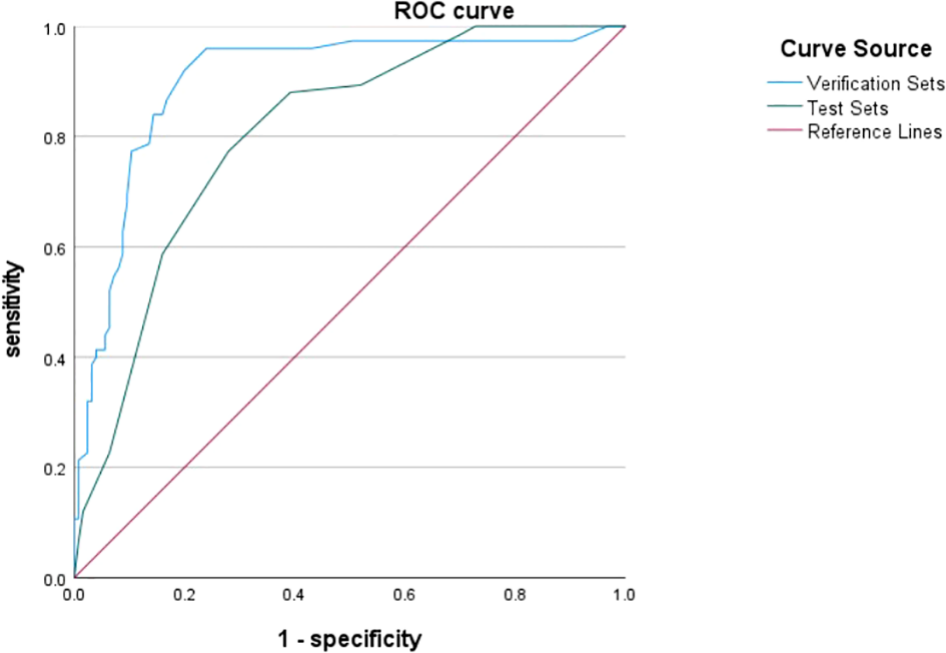
ROC curve analysis of the test and validation sets of the random forest model.

The bootstrap self-help method was used to create a random forest training model in 200 cases of study subjects and validated on the validation set. Through the parameter settings, the algorithm was found to be the best predictor when ntree=500, mtry=5 with an accuracy of 72.0%, sensitivity of 92.0%, and specificity of 80.0%. The area under the curve for the test set and validation set is 0.805 and 0.901 respectively, and the reliability and generalizability of the model have been demonstrated (**Figure 2**).

### CIPN forest map of NPC patients based on random forest algorithm and the importance ranking of variables

**Figure 3:**
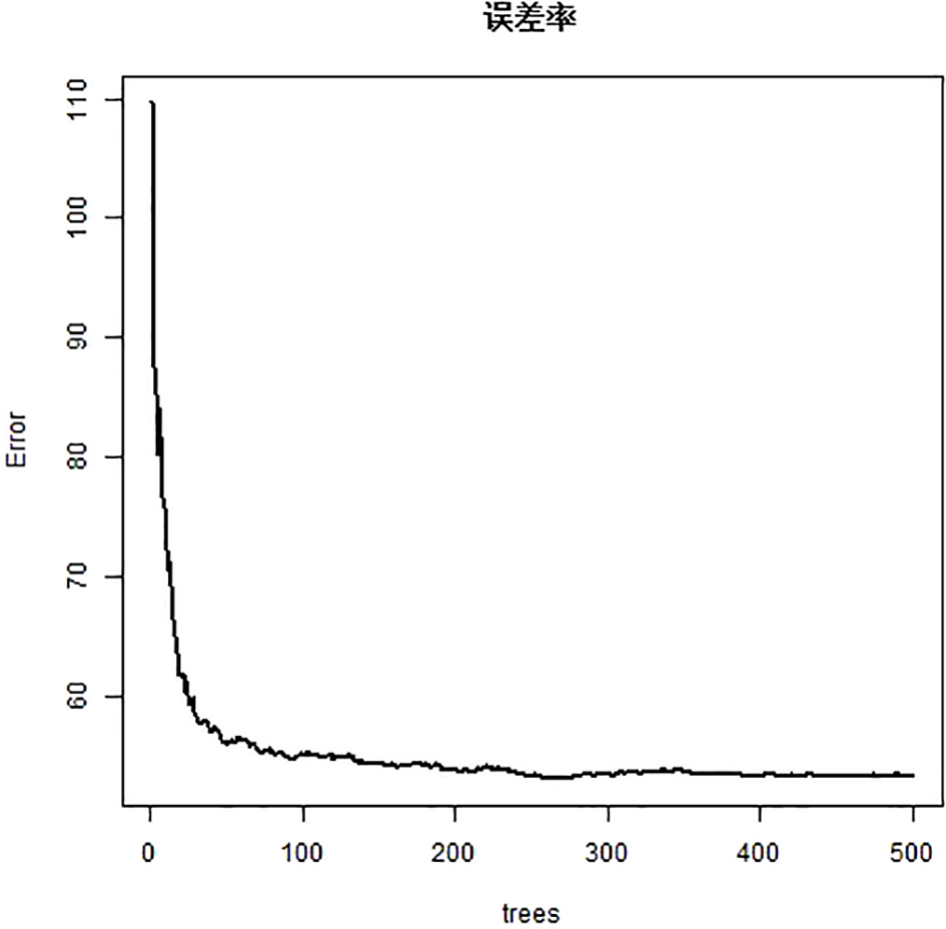
Relationship between the number of decision trees and error rate.

**Figure 4:**
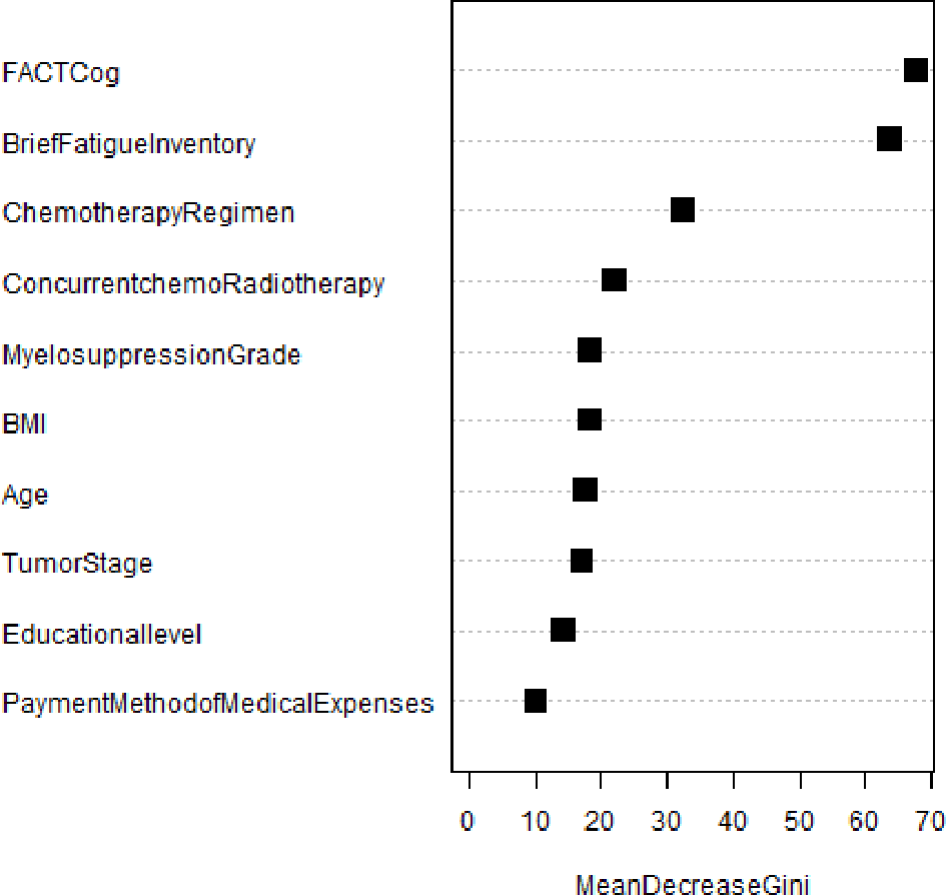
Importance ranking of variables.

**Figure 3** shows the relationship between the number of decision trees and the error rate of the random forest model. Random forest algorithm was used to rank the importance of the 17 variables initially included: (the variables with contribution rate >5%) were fact COG (57.6%), BFI-C (55.6%), HFS grade (40.5%), chemotherapy regimen (28.5%), concurrent chemoradiotherapy (19.9%), myelosuppression (16.1%), BMI (5.4%), tumor stage (14.9%), age (14.5%), education level (12.1%), and Payment method of medical expenses (8.4%). (See **Figure 4**).

### Binary logistic analysis of CIPN in NPC patients based on random forest model

**Figure 5:**
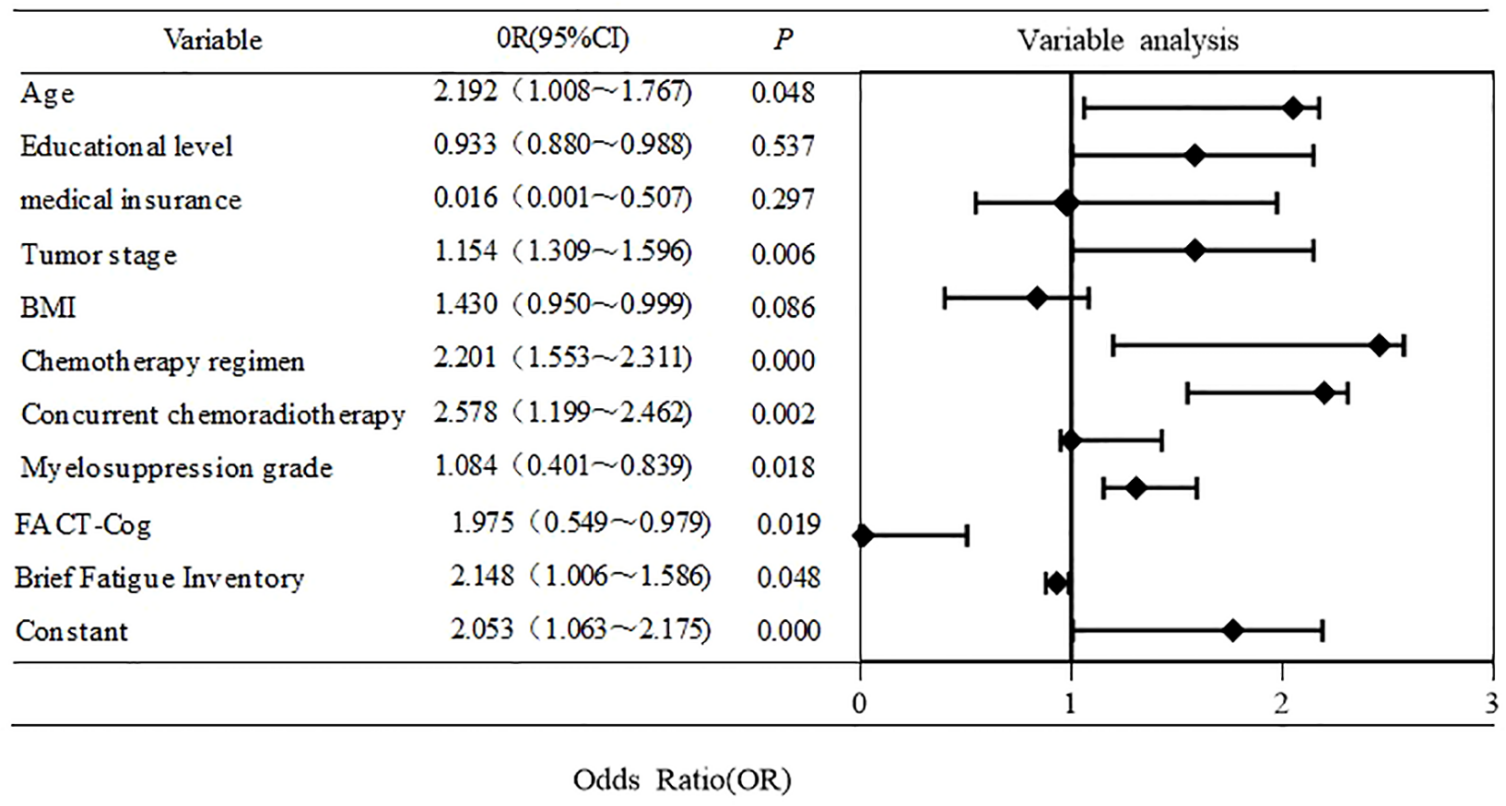
Results of random forest model analysis.

With CIPN as the dependent variable (CIPN group =1, normal group =0), the variables with a contribution rate of >5% were used as independent variables for logistic regression analysis to construct a random forest model diagram. The results showed that age, chemotherapy cycle, tumor stage, chemotherapy regimen, concurrent chemoradiotherapy, BFI-c, HFS, and fact cog were the main influencing factors of CIPN in NPC patients (*P* < 0.05). as shown in **Figure 5**.

## Discussion

### The incidence of CIPN was higher in NPC patients

Neurological complications such as numbness in the hands and feet, cognitive impairment, fatigue, and other symptoms are common adverse reactions during cancer treatment, often leading to dose reduction, prolonged treatment planning, or delayed administration, seriously affecting the treatment effectiveness and quality of life of patients. The results of this study showed that the total FACT/GOG-Ntx score of NPC patients was 22 (15, 28), with an incidence rate as high as 81.7%, which was slightly higher than that of breast cancer patients; therefore, increased attention and accurate assessment of CIPN symptoms in NPC patients are of great clinical significance for further exploring safe and effective CIPN interventions (4).

### Influencing factors of CIPN symptoms in NPC patients

#### Age, BMI

Notably, this study showed that age is one of the main influencing factors for CIPN symptoms in NPC patients. Some studies have shown that there is a close association between age and the severity of CIPN and that the activation of microglial cell signaling by TNF-α and IL-1α to secrete neurotoxic factors is related to the increased secretion of neurotoxic factors with increasing age in the context of aging (12). In the present study, a higher proportion of NPC patients were middle-aged or older (83.7% ≥31 years). In addition, BMI is an independent risk factor for CIPN in NPC patients, which is consistent with the study by Mizrahi et al. (13), because obesity is usually associated with idiopathic neuropathy, metabolic disorders, and risk of insulin resistance, which leads to neuropathy in patients; secondly, obese patients usually need to take higher doses of chemotherapeutic drugs, which aggravates CIPN symptoms (14). Therefore, it is necessary to pay attention to NPC patients with older age and higher BMI index, encourage patients to lead a healthy lifestyle, and optimize the treatment plan if necessary to alleviate CIPN symptoms.

### Chemotherapy mode, Concurrent chemoradiotherapy

Notably, the results of this study confirm that the type of chemotherapy and synchronized radiotherapy are influential factors in the development of CIPN in patients with NPC. NPC patients have long cycles of chemotherapy, usually requiring about 3-7 cycles of chemotherapy, plus platinum combined with other combination chemotherapy is more likely to lead to the accumulation of chemotherapy drug doses compared to single chemotherapy, causing corresponding CIPN symptoms (15); in addition, radiotherapy irradiates part of the patient’s brain, causing partial damage to the cranial nerves and thus aggravating CIPN symptoms (16). This suggests that clinical medical staff must pay close attention to whether the above patients have early symptoms and signs of CIPN, and closely monitor patients’ CIPN during chemotherapy and after the start of radiotherapy, duloxetine is recommended for the treatment of CIPN (17). Non-pharmacological interventions for CIPN exist, such as exercise and behavioral interventions (18).

### ACT/GOG-Ntx and hand-foot syndrome、FACT-Cog、brief fatigue relationship

We found that Hand-foot syndrome is a dose-limiting skin toxicity that can be caused by many chemotherapeutic agents (19). Logistic regression analysis showed that Hand-Foot Syndrome, FACT-Cog, and Brief Fatigue could predict CIPN in NPC patients. Hand-foot syndrome is a dose-limiting dermal toxicity reaction, many chemotherapeutic agents can cause the occurrence of this adverse reaction, thus, hand-foot syndrome is one of the prominent symptoms of CIPN, and hand-foot syndrome symptoms the more significant patients CIPN symptoms (20). Cognitive decline is associated with CIPN, blood-brain barrier disruption, decreased neurogenesis in the hippocampus, secondary neuroinflammatory response, and oxidative stress, which further impairs patients’ neuronal functions such as perception, thinking, and memory, while neurological dysfunction further impairs patients’ cognitive functions, and the two are interrelated (21); CIPN is usually associated with sensorimotor and activity disorders, and BFI-C often leads to blunted sensory and motor functions of patients, so CIPN is also closely related to Hand-Foot Syndrome, FACT-Cog, Brief Fatigue. Therefore, clinicians need to instruct patients in skin self-management and pay attention to the skin sensation of their hands and feet (22). Therefore, it is recommended to formulate a scientific and effective exercise program according to the patient’s physical condition and gradually exercise according to the patient’s tolerance to improve neurotransmitter conduction and alleviate the symptoms of Brief Fatigue and CIPN. In addition, carrying out effective cognitive rehabilitation training and combining it with traditional Chinese medicine acupuncture treatment to stimulate the nervous system has certain clinical value for improving the overall cognitive function of NPC patients and alleviating CIPN symptoms.

## Conclusion and prospect

In conclusion, the incidence of CIPN in NPC patients is high and affected by many factors, random forest model-based algorithms can effectively predict and identify important variable factors in CIPN symptoms, but there is no effective treatment plan at present. This study is a single center, cross-sectional investigation and study, and the representation of the research object is limited. In the future, clinicians can reduce study bias by incorporating random forest algorithms, using more objective measures of CIPN symptoms, cognitive assessments, and other measures.

## Data Availability

The original contributions presented in the study are included in the article/supplementary material. Further inquiries can be directed to the corresponding author.
